# Identification of novel biomarkers to monitor β-cell function and enable early detection of type 2 diabetes risk

**DOI:** 10.1371/journal.pone.0182932

**Published:** 2017-08-28

**Authors:** Kirstine J. Belongie, Ele Ferrannini, Kjell Johnson, Patricia Andrade-Gordon, Michael K. Hansen, John R. Petrie

**Affiliations:** 1 Cardiovascular and Metabolic Disease Research, Janssen Research & Development, Spring House, Pennsylvania, United States of America; 2 CNR Institute of Clinical Physiology, Pisa, Italy; 3 Arbor Analytics, Ann Arbor, Michigan, United States of America; 4 Institute of Cardiovascular and Medical Sciences, University of Glasgow, Glasgow, United Kingdom; Broad Institute, UNITED STATES

## Abstract

A decline in β-cell function is a prerequisite for the development of type 2 diabetes, yet the level of β-cell function in individuals at risk of the condition is rarely measured. This is due, in part, to the fact that current methods for assessing β-cell function are inaccurate, prone to error, labor-intensive, or affected by glucose-lowering therapy. The aim of the current study was to identify novel circulating biomarkers to monitor β-cell function and to identify individuals at high risk of developing β-cell dysfunction. In a nested case-control study from the Relationship between Insulin Sensitivity and Cardiovascular disease (RISC) cohort (n = 1157), proteomics and miRNA profiling were performed on fasting plasma samples from 43 individuals who progressed to impaired glucose tolerance (IGT) and 43 controls who maintained normal glucose tolerance (NGT) over three years. Groups were matched at baseline for age, gender, body mass index (BMI), insulin sensitivity (euglycemic clamp) and β-cell glucose sensitivity (mathematical modeling). Proteomic profiling was performed using the SomaLogic platform (Colorado, USA); miRNA expression was performed using a modified RT-PCR protocol (Regulus Therapeutics, California, USA). Results showed differentially expressed proteins and miRNAs including some with known links to type 2 diabetes, such as adiponectin, but also novel biomarkers and pathways. In cross sectional analysis at year 3, the top differentially expressed biomarkers in people with IGT/ reduced β-cell glucose sensitivity were adiponectin, alpha1-antitrypsin (known to regulate adiponectin levels), endocan, miR-181a, miR-342, and miR-323. At baseline, adiponectin, cathepsin D and NCAM.L1 (proteins expressed by pancreatic β-cells) were significantly lower in those that progressed to IGT. Many of the novel prognostic biomarker candidates were within the epithelial-mesenchymal transition (EMT) pathway: for example, Noggin, DLL4 and miR-181a. Further validation studies are required in additional clinical cohorts and in patients with type 2 diabetes, but these results identify novel pathways and biomarkers that may have utility in monitoring β-cell function and/ or predicting future decline, allowing more targeted efforts to prevent and intercept type 2 diabetes.

## Introduction

Type 2 Diabetes is a heterogeneous disease caused by insulin resistance, β-cell insufficiency or a combination of both but the specific pathophysiology of the disease in the individual patient is usually unknown.

Currently, methods of measuring β-cell function are inaccurate, prone to error, labor-intensive, or affected by glucose-lowering therapy. For example, in the ADOPT study, the β-cell function parameter HOMA-B was biased by the acute effects of glyburide, a sulfonylurea that increases insulin secretion, in that a large apparent “improvement” in β-cell function in the first year was followed by a sharp decline. During years 4 and 5 of the study, overall HbA_1c_ deteriorated much faster with glyburide than with metformin or rosiglitazone therapy while HOMA-B remained stable [1].

Progression to type 2 diabetes is associated with a decline in β-cell function usually on a background of long-standing insulin resistance [2–5]. Using current methodology it is not possible to identify with an acceptable degree of certainty which prediabetic individuals have the highest risk of progression. Whether they are classified as having impaired fasting glucose (IFG) or impaired glucose tolerance (IGT), such individual have a definite impairment in β-cell function, detected as a reduced acute insulin response to intravenous glucose or reduced insulinogenic index in an oral glucose tolerance test (OGTT) [6, 7].

An important index of β-cell function is β-cell glucose sensitivity (the change in insulin secretion rate for any given change in plasma glucose concentration), calculated by mathematical modeling of C-peptide concentrations following an oral glucose load [8]. This parameter tracks quite accurately with β-cell function in longitudinal [9] as well as intervention studies [10]; however, the requirement for a glucose challenge and multiple blood draws over a two-hour period make it unsuitable for population studies or large scale trials of new drugs.

Novel prognostic biomarkers are therefore required to identify individuals at high risk of decline in β-cell function. A validated set of novel biomarkers could enhance the accuracy of disease prediction, provide novel insights into pathophysiology and contribute to future prevention and interception of cases of the type 2 diabetes.

In the current study, we performed an unbiased screen of proteins and miRNAs in an established longitudinal cohort (RISC) in which both insulin sensitivity and β-cell function were well characterized at baseline [9, 11]. We compared individuals who developed IGT and exhibited a decline in β-cell glucose sensitivity over three years of observational follow up with those who remained NGT with stable β-cell glucose sensitivity. The aim was to identify proteins and miRNAs that could potentially track β-cell function and predict future changes in β-cell function.

## Materials and methods

### Cohort

We studied samples from the European RISC (Relationship between Insulin Sensitivity and Cardiovascular Disease) cohort [7, 9, 11]. In brief, RISC is a longitudinal study of nondiabetic, normotensive adults (n = 1384) characterized for both insulin sensitivity (hyperinsulinemic euglycemic clamp) and β-cell function (multiple sampling OGTT with mathematical modelling) at baseline. Written consent was provided by all participants for their information to be stored in the RISC database and used for research purposes, including exploratory biomarker work. The study underwent appropriate review by the European Commission research program and its ethics committee. The current retrospective analysis described did not require additional review due to prior approval of future biomedical analyses at the time of initiation.

At follow-up (3 years), multiple sampling OGTTs with mathematical modelling were completed, but the hyperinsulinemic euglycemic clamp was not repeated and insulin sensitivity was calculated from the OGTT-derived parameter Oral Glucose Insulin Sensitivity index, (OGIS) [12]. Among participants with normal glucose tolerance (NGT) at baseline (n = 1,157), 8% (n = 90) developed IGT at follow-up, which was associated with an average 40% decline in β-cell glucose sensitivity and a 30% decline in insulin sensitivity. For our study, we selected 43 IGT individuals with the largest decline in β-cell glucose sensitivity at follow-up (cases) and 43 individuals experiencing no decline in β-cell glucose sensitivity (controls). Both groups were matched at baseline for age, gender, BMI, waist-hip ratio, insulin sensitivity, family history of diabetes, β-cell glucose sensitivity (Table 1).

**Table 1:** Demographic and clinical characteristics of the RISC cohort*.

	Baseline		*p*-value	Follow-up		*p*-value
	Controls	Cases		Controls	Cases	
**NGT/IGT**	43/0	43/0	-	43/0	0/43	-
**Familial diabetes**	16/43	12/43	0.359	17/37	14/35	0.610
**Age (years)**	45.2 ± 7.5	44.8 ± 8.0	0.813			
**Sex (M/F)**	17/26	18/25				
**BMI (kg/m**^**2**^**)**	26.2 ± 3.7	26.2 ± 4.0	0.955	26.5 ± 3.6	26.8 ± 4.6	0.692
**Waist-hip ratio**	0.87 ± 0.07	0.87 ± 0.10	0.645	0.90 ± 0.07	0.90 ± 0.08	
**Fasting [G] (mmol/L)**	4.93 ± 0.43	5.05 ± 0.36	0.186	5.13 ± 0.70	5.42 ± 0.53	**0.036**
**2-hr [G] (mmol/L)**	5.82 +/-1.00	6.20 +/-0.92	0.071	5.46 +/-1.12	8.57 +/-0.82	**<0.001**
**Fasting [I] (pmol/L)**	35 ± 18	39 ± 19	0.373	39 ± 19	49 ± 39	0.166
**2-hr [I] (pmol/L)**	196 ± 136	250 ± 244	0.248	195 ± 136	438 ± 378	**<0.001**
**Insulin sensitivity (M)**	114 ± 43	113 ± 56	0.906	ND	ND	-
**OGIS (ml**^**.**^**min**^**-1.**^**kg**^**-1**^**)**	11.1 ± 2.3	10.9 ± 1.9	0.666	11.6 ± 2.6	8.7 ± 2.6	**<0.001**
**Glucose sensitivity**	110 ± 41	110 ± 47	0.943	151 ± 74	83 ± 30	0.0000

* Entries are mean ± SD. [G] = plasma glucose concentration; [I] = plasma insulin concentration.

### Proteomics

1,129 proteins were measured from 75 μl of plasma using the multiplexed SomaLogic technology according to SomaLogic’s proprietary method [13]. Briefly, DNA aptamers called SOMAmers (slow off-rate modified aptamers) were developed for each of the 1,129 proteins and quantified using DNA microarray methodology. SOMAmers are protein recognition reagents with high binding affinities and stable chemical structures. The plasma samples were incubated with a mixture of SOMAmers to generate SOMAmer-protein complexes. Samples were then cleared of unbound SOMAmers and proteins by two steps of stringent washing and bead-based immobilization. SOMAmers were then eluted under high pH-denaturing conditions and quantified by hybridizing to a DNA microarray. The SOMAmer mixture quantitatively reflects the original protein concentration and results were provided in relative fluorescence units (RFU).

### miRNA profiling

RT-PCR based miRNA high-throughput detection according to Regulus Therapeutics’ proprietary method was used (http://regulusrx.com/) to measure 754 micro-RNAs. Briefly, 200 μl of plasma is added to Qiagen spin columns and after an initial QC using specific miRNAs, the solution is reverse transcribed using Megaplex RT primers, cDNA is amplified with Megaplex preamp primers and loaded onto Open Array microRNA card and run using QuantStudio Real Time PCR system. Data quality control and filtering were done by Regulus; for example, miRNA assays with less than 20% detected values across samples were excluded. This left 203 miRNA for further analysis with values log-transformed to account for their non-normal distribution.

### Statistical analysis

Potential relationships with classification status, univariate statistics were initially calculated between each predictor and responder classification. Specifically, the predictors were ranked on their ability to distinguish cases from controls in order of ascending *p*-values on the basis of unequal variance *t*-tests. While this approach can identify individual predictors that are associated with an outcome, it has the weakness of missing sets of predictors that may work in conjunction with each other for outcome classification. A predictive modeling approach was therefore used for predicting outcome classification.

Prior to modeling the data, several pre-processing steps were taken to avoid characteristics that lead to model failure. These included the removal of near-zero variance predictors, removal of highly correlated predictors, mean-centering and scaling of each predictor, and imputation of missing predictor values as described in Kuhn and Johnson (2013) [14]. Several predictive models were built to uncover relationships between predictors and cases and controls. The models used for this data were: partial least squares, support vector machines, recursive partitioning, and random forests. These models were specifically selected to be able to uncover linear or complex non-linear separation between the outcome classes. Each of these models has one or more tuning parameters. To select the optimal value of the tuning parameters and to assess model classification performance, 5 repeats of 10-fold cross-validation were used. Furthermore, because there were many more predictors than samples, recursive feature elimination was used to eliminate predictors that were not associated with classification status [14]. Recursive feature elimination prevents over-fitting to the data and selects only those predictors that are important for true hold-out sets.

The rank ordering of strength of each of the predictors for each of the models was aggregated across models to generate a consensus view of predictor importance. We combined predictor importance information with the univariate statistics to identify predictors that were not independently useful, but appeared to have utility when viewed in conjunction with other predictors.

All relevant data are within the paper and its Supporting Information files.

## Results

At baseline, cases and controls were well-matched for all anthropometric, clinical, and metabolic characteristics (Table 1). At year 3, by design, cases had higher 2-hr OGTT plasma glucose and lower β-cell glucose sensitivity; they also had higher fasting plasma glucose levels, higher insulin concentrations, and significantly lower insulin sensitivity.

### Diagnostic protein biomarkers of β-cell dysfunction

**Fig 1:**
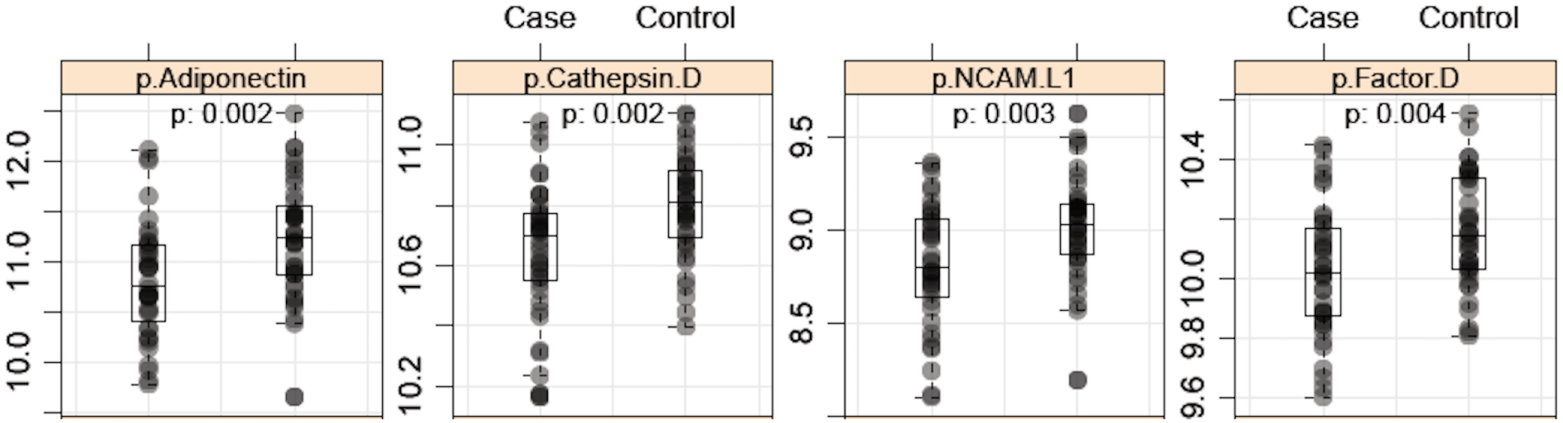
Scatterplot of diagnostic protein biomarkers. Circulating levels of top-ranked diagnostic biomarkers in IGT subjects (case, left) compared to healthy controls (right).

Cross-sectional analysis was performed at the 3-year time-point to identify differentially expressed proteins reflective of current β-cell function. Following quality control procedures, Somalogic data were available from 40 cases and 40 controls for 1,025 proteins. Univariate statistics identified 41 proteins differentially expressed (p<0.05) between cases and controls at year 3 (Table 2 and S1 Table). In addition, a multivariate classification approach was used to identify predictors that best separated cases from controls. Fourteen proteins were identified by all four predictive models; another 18 were identified by three of the four models. Low adiponectin, a well-known biomarker linked with insulin resistance and β-cell function [15–18], was a top diagnostic protein reflecting decline in β-cell glucose sensitivity by both multivariate and univariate analysis (Fig 1). Histidine-rich glycoprotein (HRG), also highly ranked and identified with lower plasma levels in cases in our analysis, is complexed with adiponectin in human serum [19]. Endocan, a dermatan sulfate proteoglycan expressed in endothelial cells and adipocytes and usually associated with inflammation [20], was highly ranked with decreased plasma levels in cases at both baseline and at follow-up. Other proteins involved in inflammation such as sTNFR2 (increased), CD30 (TNF family, decreased), and kallikrein 5 (increased) were also differentially expressed. Carbonic anhydrase 3 (CA3) was highly ranked in multivariate analysis, although only borderline significant in the univariate analysis with reduced protein levels in cases. CA3 catalyzes the reversible hydration of carbon dioxide to bicarbonate and is abundantly found in adipocytes, muscle, and liver [21].

**Table 2:** Diagnostic predictors of β-cell function*.

Proteins				miRNAs			
Univariate	FC	Multivariate	FC	Univariate	FC	Multivariate	FC
Adiponectin	0.76	FCG2A/B	1.44	miR-181a	0.18	miR-181a	0.18
kallikrein.5	1.14	Adiponectin	0.76	miR-323-3p	0.36	miR-323-3p	0.36
CHRDL1	0.88	Carbonic anhydrase III	1.38	miR-222	0.12	miR-342-3p	0.22
Endocan	0.85	sLeptin R	1.20	miR-483-5p	0.31	miR-151-5P	0.44
K.ras	1.14	Endocan	0.85	miR-454	0.34	miR-330	0.34
HRG	0.85	KI3S1	1.35	miR-151-5P	0.44	miR-454	0.34
a1.Antitrypsin	0.86	HRG	0.85	miR-330	0.34	miR-212	0.38
FCG2A.B	1.44	kallikrein 5	1.14	miR-652	0.23	miR-451	2.02
G.CSF.R	0.86	K-ras	1.14	miR-532-3p	0.46	miR-483-5p	0.31
Cystatin.M	0.85	CRDL1	0.88	miR-212	0.38	miR-136-star	0.55
IFN.lambda.2	0.86	NCAM-L1	0.91	miR-342-3p	0.22	miR-532-3p	0.46
Layilin	0.90	FCG3B	1.12	miR-136-star	0.55	miR-636	3.34
KI3S1	1.35	NRP1	0.92	miR-142-5p	0.52	miR-7	1.03
Cadherin.12	0.74	TNF sR-II	1.08	miR-433	0.52	miR-625-star	0.40
IGFBP-1	0.72	Cadherin-12	0.74	miR-204	0.56	miR-215	0.32
Aminoacylase.1	1.36	p27Kip1	1.33	miR-432	0.48	*miR-432*	*0*.*48*
Angiopoietin.4	0.90	IL-1b	0.84	miR-625-star	0.40	*miR-30a-5p*	*1*.*00*
RUXF	1.12	Carbonic anhydrase V	0.75	miR-451	2.02	*miR-134*	*0*.*49*
Kallikrein.4	0.89	HIPK3	1.24	miR-636	3.34	*miR-30d*	*0*.*92*
OSM	1.11	NKp30	1.25	miR-27b	0.26	*miR-16*	*1*.*38*

* Fold change (FC) is calculated as Case—Control. Entries in italics: Identified in 2 out of 4 predictive models.

### Diagnostic miRNA biomarkers of β-cell dysfunction

miRNAs are important regulators of protein expression and are valuable as biomarkers due to their stability in plasma. At year 3 in cross-sectional analysis, data were available from 40 cases and 40 controls: 27 miRNAs were differentially expressed using univariate statistics (p≤0.05) in cases vs. controls (Table 2 and S1 Table). Using multivariate analysis, 6 miRNAs were identified in all four predictive models and 9 miRNAs were identified in three models (S1 Table). Thirteen of the 15 top diagnostic miRNAs identified in multivariate testing were in common with the univariate list.

Full data were available from 38 cases and 38 controls for all measured protein biomarkers and miRNAs for combined analysis with clinical covariates. Predictive models identified the β-cell function parameter glucose during OGTT (gluO120, gluO90, gluO60), as the top predictor (S3 Table). The top ranked non-OGTT predictors across the multivariate models were adiponectin, kallikrein 5, CA3, sTNFR2, cadherin12, endocan, CRDL1 and miR-181a among others.

### Prognostic protein biomarkers of β-cell dysfunction

**Fig 2:**
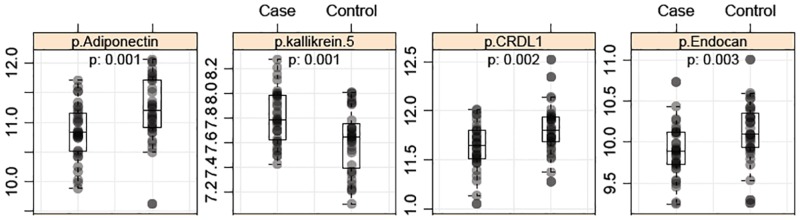
Scatterplot of prognostic protein biomarkers. Circulating levels of top-ranked prognostic biomarkers in IGT subjects (case, left) compared to healthy controls (right).

Data were available at baseline and follow-up from 36 cases and 36 controls. Univariate statistics identified 60 proteins differentially expressed (p≤0.05) between cases and controls at baseline (Table 3 and S2 Table). A multivariate analysis with recursive feature elimination using several different statistical methods allowed for the ranking of these proteins according to importance. This analysis resulted in four proteins (adiponectin, noggin, DLL4, sialoadhesin) ranking as important across all four models, while another 26 proteins were highly ranked in three out of four models (Table 3 and S2 Table). We identified several proteins relevant to diabetes among the top ranked proteins at baseline. Adiponectin, was the highest ranked biomarker (by both multivariate and univariate analysis) of decline in β-cell glucose sensitivity (Fig 2). Cathepsin D was also a top ranked predictor in both analyses, with protein levels reduced in cases. Cathepsin D was shown in a recent prospective study using two other cohorts to have a strong association with insulin resistance [22]. Other well-known diabetes-associated molecules such as fibrinogen, IL-1 β and IL-6 were among other very highly ranked predictors. NCAM-L1, Factor D (adipsin), CDK5-p35, endocan, noggin, DLL4 were identified as novel potential prognostic biomarkers of β-cell function and were among the top ranked proteins.

**Table 3:** Prognostic predictors of β-cell function*.

Proteins				miRNAs			
Univariate	FC	Multivariate	FC	Univariate	FC	Multivariate	FC
Adiponectin	0.75	Adiponectin	0.75	miR-342-3p	.25	miR-181a	0.21
Cathepsin.D	0.91	Noggin	0.82	miR-181a	0.21	miR-590-3P	0.55
NCAM.L1	0.87	DLL4	0.83	miR-590-3P	0.55	miR-27b	0.26
Factor.D	0.91	Sialoadhesin	1.20	miR-497	1.49	miR-222	0.23
CDK5.p35	0.91	CDK5/p35	0.91	miR-25	0.14	miR-323-3p	0.46
Endocan	0.85	COLEC12	0.89	miR-323-3p	0.46	miR-151-5P	0.53
Coagulation.Factor.XI	0.89	FGF-12	1.08	miR-151-5P	0.53	miR-142-5p	0.61
Cystatin.M	0.84	Cathepsin D	0.91	let-7c	0.67	miR-625-star	0.42
MIS	0.90	P-Cadherin	0.91	miR-27b	0.26	miR-205	0.66
Fibrinogen	0.91	NCAM-L1	0.87	miR-483-5p	0.37	*miR-342-3p*	*0*.*25*
CHL1	0.91	C1QBP	0.93	miR-625-star	0.42	*miR-223*	*0*.*11*
SCF.sR	0.89	TNFSF18	0.96	miR-205	0.66	*miR-331*	*1*.*41*
Carbonic.Anhydrase.IV	0.91	Coagulation Factor XI	0.89	miR-532-3p	0.53	*miR-497*	*1*.*49*
MM1	1.39	TCCR	0.89	miR-375	0.53	*let-7g*	*0*.*39*
PAK3	0.92	IL-11	0.87	miR-223	0.11	*miR-211*	*0*.*56*
ARGI1	0.93	Layilin	0.91	miR-222	0.23	*miR-652*	*0*.*38*
Noggin	0.82	IL-1b	0.80	miR-142-5p	0.61	*miR-375*	*0*.*53*
GDF2	0.77	Endocan	0.85			*miR-125b*	*0*.*83*
DLL4	0.83	Factor D	0.91			*miR-378-1*	*1*.*52*
IL.6	1.27	Semaphorin 3A	0.83			*miR-532-3p*	*0*.*53*

* Fold change (FC) is calculated as Case—Control. Entries in italics: Identified in 2 out of 4 predictive models.

A pathway analysis of baseline proteins with *p*-values < 0.05 using GeneGo (https://portal.genego.com/) identified the epithelial-mesenchymal-transition (EMT) pathway as an important differentially regulated pathway in cases vs. controls, and included proteins such as noggin, DLL4, FGF12, GDF2 (BMP-9), and E-cadherin. Further down the prognostic list was IGFBP-2, whose circulating levels were lower in cases at baseline. In men, low levels of IGFBP-2 have been reported to be associated with metabolic syndrome [23].

### Prognostic miRNA biomarkers of β-cell dysfunction

Data were available at baseline and follow-up from 40 cases and 40 controls. Differential expression of 17 miRNAs with *p*-values at or below 0.05 in an unequal variance *t*-test comparing controls to cases at baseline was identified (Table 3). Individuals that progressed to prediabetes and reduced β-cell function exhibited a decrease in 16 of these 17 miRNAs, while miR-497 was increased. Top differentially expressed miRNAs included miR-342-3p, miR-181a, miR-590-3p, miR-497, and miR-25, whereas most abundant differentially expressed miRNAs were miR-223, miR-483-5p, miR-375, miR-27b, and miR-222. Multivariate tests with four predictive models identified two miRNAs (miR-181a and miR-590-3p) as important across all four models with nine miRNAs in total identified as important in at least three of these. All of these miRNAs were common between the univariate and multivariate list but the rank ordering was different. miR-27b, miR-222 and miR-142-5p, among others, were ranked higher in the multivariate list.

## Prognostic protein and miRNA biomarkers of β-cell dysfunction

Combined data were available from 35 cases and 35 controls for all measured protein biomarkers and miRNAs for combined analysis with clinical covariates Predictive models identified 2-hr plasma insulin levels and potentiation as the highest rank predictors of future β-cell function (S3 Table). Potentiation is a time-dependent factor that characterizes an aspect of β-cell function aspect distinct from β-cell glucose sensitivity [24]. The top protein predictors were adiponectin and then FGF12, cathepsin D, STK16, DLL4 and CD30. The top miRNA biomarkers were miR-342-3p, miR-181a, and miR-590-3p (S3 Table). The top prognostic biomarkers between the univariate analysis and multivariate analyses were very similar with mainly differences in the rank order.

### Overlap of diagnostic and prognostic biomarkers

**Fig 3:**
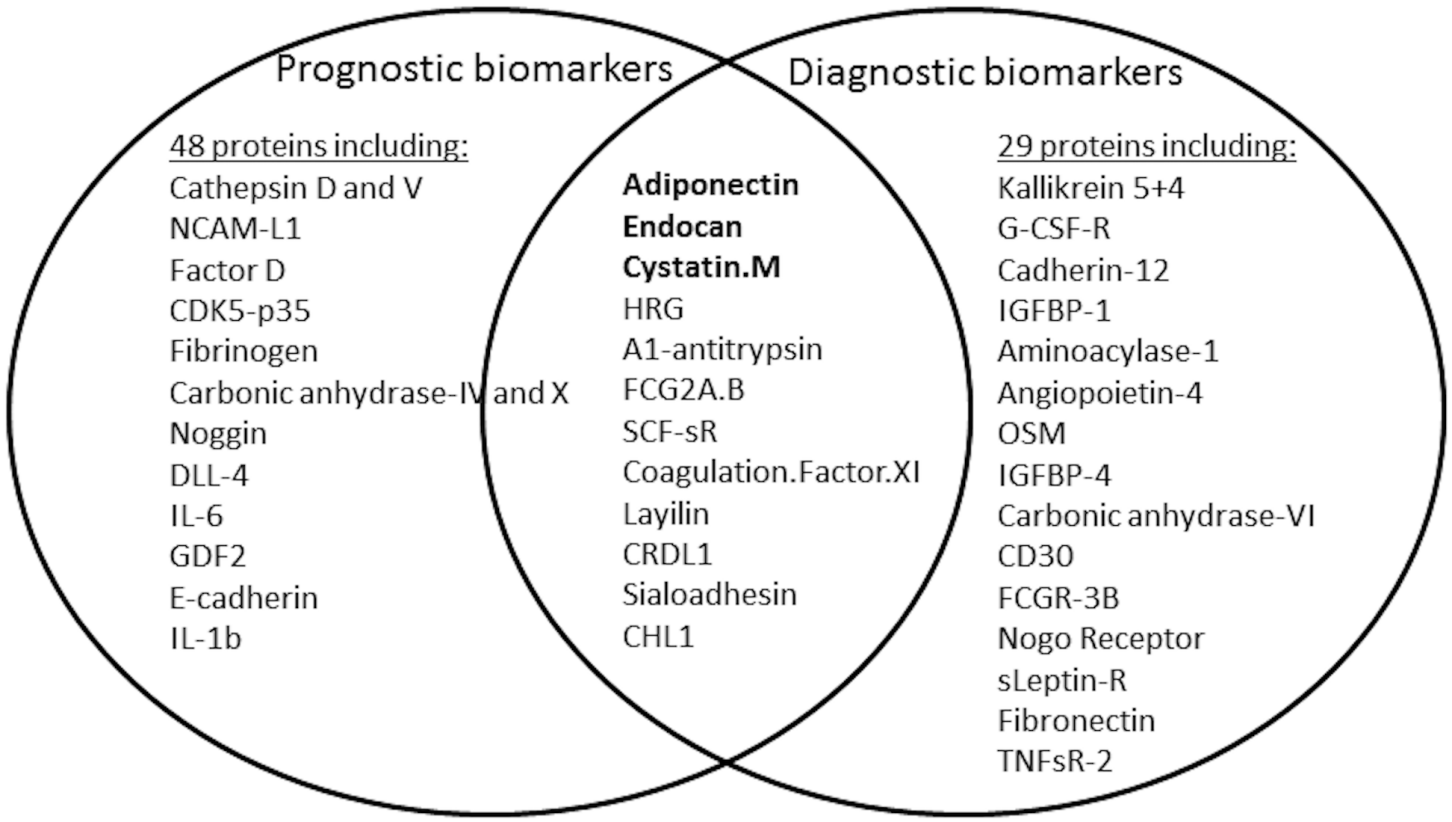
Venn diagram of prognostic and diagnostic protein predictors based on the univariate analysis with p<0.05. Twelve proteins were identified as differentially expressed at both time-points; 48 proteins were only predictive at baseline; and 29 only at follow-up. Proteins in bold were among top 20 in both prognostic and diagnostic lists.

**Fig 4:**
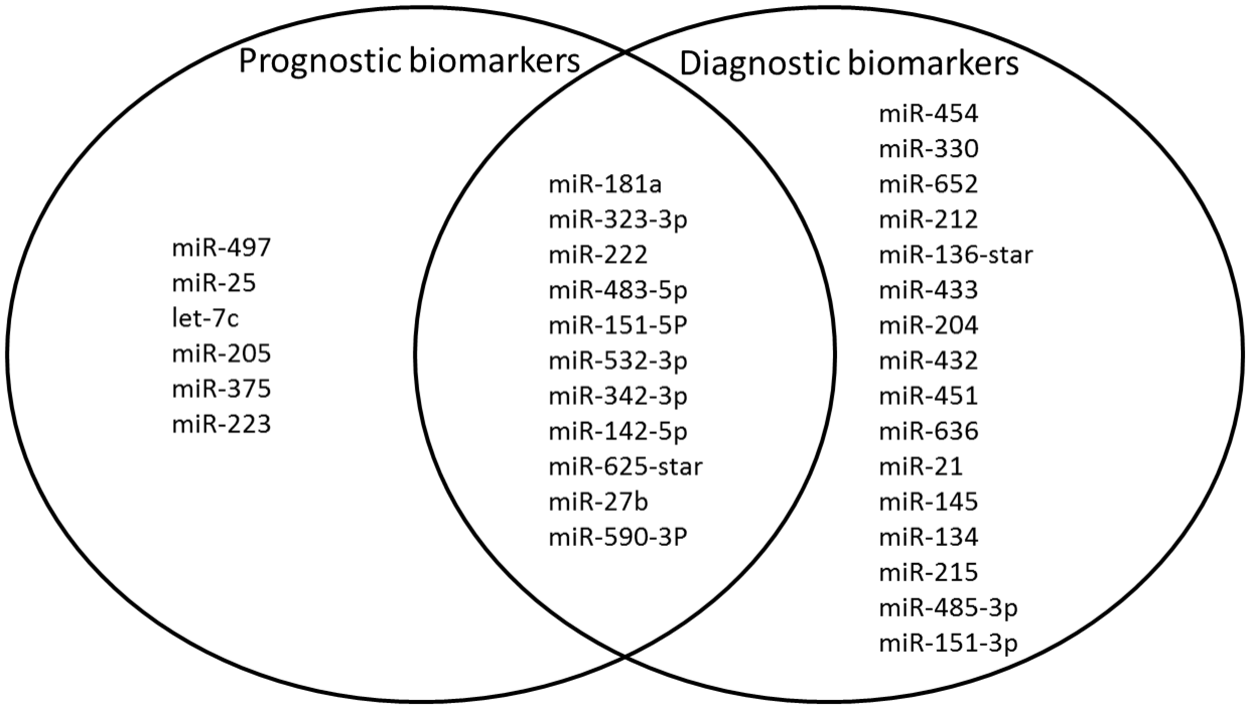
Venn diagram of prognostic and diagnostic miRNA predictors based on the univariate analysis with p<0.05. 11 miRNAs were identified as differentially expressed at both time-points; 6 miRNAs were predictive of ß-cell glucose sensitivity at baseline only; and 16 only at follow-up.

While only 12 of 77 protein biomarkers (16%) identified in the diagnostic list (univariate) were also differentially expressed at baseline and identified as prognostic biomarkers (Fig 3), this was the case for 50% of the top 20 miRNAs (Fig 4).; For example, the four top miRNAs from the diagnostic (multivariate) list (miR-181a, miR-323-3p, miR-342-3p, and miR-151-5p) were also differentially expressed at baseline. These miRNAs are respectively involved in hepatic insulin sensitivity (miR-181a) [25], expressed in human β-cells (miR-323-3p) [26], identified in PBMCs from people with diabetes (miR-342-3p) [27], and regulated by exercise (miR-151-5p) [28]. At follow-up, miR-330, miR-454, and miR-212 among others were selectively changed (Fig 4). Of the 12 proteins differentially expressed at both baseline and follow-up, six (adiponectin, endocan, coagulation factor XI, cystatin M, CHL1, and SCF-sR) were in the top 20 prognostic list and seven (adiponectin, CRDL1, endocan, HRG, alpha1-antitrypsin, FCG2A/B, and cystatin M) were in the top 20 of the univariate diagnostic list (Fig 3). Five proteins were found in both the multivariate prognostic and diagnostic lists; adiponectin, sialoadhesin, NCAM-L1, IL-1 β and endocan (Tables 2 and 3).

## Discussion

The current study used plasma proteomics and miRNA profiling in samples obtained from the longitudinal RISC study to identify prognostic and diagnostic protein and miRNA biomarkers predicting or reflecting β-cell glucose sensitivity. Several protein biomarkers were identified reflecting decline in β-cell glucose sensitivity at follow-up that also showed value to distinguish cases from controls at baseline, even although groups were well matched for glucose tolerance, insulin sensitivity, and β-cell glucose sensitivity (as well as standard clinical covariates). At the top of the list of proteins identified, adiponectin, endocan, and sialoadhesin were of particular interest as they show potential value in both monitoring and predicting future decline in β-cell function. However, a novel finding was the implication of both proteins and miRNAs within the EMT pathway as differentially expressed at baseline in those that progressed to IGT and reduced β-cell function. EMT is, among other functions, prominent during formation of endocrine cells in the pancreas in embryogenesis [29].

Adiponectin is known to regulate glucose levels and fatty acid breakdown and to enhance insulin sensitivity in muscle and liver [30]. Our results implicate low baseline plasma adiponectin levels in healthy individuals as a predictor of future β-cell dysfunction and IGT. We observed a 0.74-fold decrease in plasma adiponectin in individuals who progressed to worse β-cell glucose sensitivity and IGT. These data are corroborated by a recent longitudinal study in which individuals who progressed from NGT to prediabetes/diabetes had lower baseline adiponectin (8.4±4.0 μg/mL) compared to non-progressors (9.9±5.8 μg/mL) [31]. Another recent study in non-obese individuals at risk for diabetes demonstrated a negative association between baseline adiponectin and change in 2-hr plasma glucose, fasting insulin, HOMA-IR and HOMA- β [32]. In a further study, the resistin-adiponectin ratio (RA index) was strongly associated with β-cell function—assessed by OGTT-derived indices—in two NGT cohorts with different degrees of overweight/obesity [33]. The authors suggested that waist-hip ratio is a strong modulator of β-cell function [33] but in the present study neither waist-hip ratio nor resistin had predictive value. Previous analyses of the RISC cohort using 1017 baseline samples showed adiponectin levels were positively associated with insulin sensitivity and HDL-cholesterol, partly explaining how low plasma concentrations of adiponectin predict insulin resistance [15, 16]. Alpha1-antitrypsin (α1-AT), another candidate biomarker found in our studies with lower plasma levels in cases both at baseline and follow-up, is a serine proteinase inhibitor associated with type 2 diabetes [34–36] which amongst other effects inhibits the enzymatic activity of neutrophil elastase (NE) (which degrades adiponectin) and cathepsin G.

Several of the top prognostic proteins and miRNA we identified are functional in pancreatic islets [37]. In addition, our results are in keeping with those of a very recent study describing cathepsin D as a marker of insulin resistance [22]; moreover, cathepsin D expression is reduced in islets from people with type 2 diabetes [38]. CDK5 was another islet protein identified in our proteomics screen to predict β-cell dysfunction: when over-activated by glucose it suppresses insulin gene expression [39, 40]. Conversely, decreased CDK5 activity enhances glucose-stimulated insulin secretion (GSIS) [40]. With regard to the top 20 miRNAs we identified, both miR-27b and miR-375 are abundant in human islets [41–43]. ADIPOR2 is a confirmed target for miR-375 [44] and miR-323-3p is enriched in human β-cells compared to α-cells [26]. miR-212 was expressed at follow-up but not at baseline. Silencing of miR-212 reduced, and overexpression increased, GSIS [45]; moreover, miR-212 was significantly up-regulated by GLP-1 (>2-fold) in isolated rat, mouse, and human islets [46].

Noggin, which was lower in cases *vs* controls, is a bone morphogenetic protein (BMP) antagonist shown to induce differentiation of embryonic stem cells into pancreatic and endocrine lineages [47]. DLL4 (Delta-like ligand 4) is a Notch ligand that suppresses the Delta-Notch signaling pathway and is permissive for pancreatic endocrine development [48]. GDF2 (BMP9 is the protein encoded by GDF2), which was high on the list of proteins predictive of decline in β-cell glucose sensitivity, has been shown to improve glucose homeostasis in both diabetic and nondiabetic rodents by acting on hepatocytes and myotubes [49, 50]. P-cadherin and E-cadherin, both lower in cases than controls, are modulated during EMT when epithelial cells lose their characteristics [51]. NCAM-L1, also lower in cases, can remodel extracellular matrix and has been found to be elevated in type 2 diabetes people with retinopathy [52].

An unexpected number of prominent prognostic predictors were in the EMT pathway, a conserved pathway in which epithelial cells lose their characteristics and start migrating as mesenchymal cells [29]. During EMT, tissue is remodelled via highly regulated gene expression such that epithelial cells lose their cell-cell junctions, apical-basal polarity and re-organize their cytoskeleton. Our data implicate proteins in the EMT pathway as novel potential prognostic and insulin-independent biomarkers for predicting decline in human β-cell function. miRNAs with known functions in the EMT pathway were also differentially expressed at baseline and correlated with a change in β-cell glucose sensitivity: miR-181a, which was top ranking at both baseline and follow-up, has been shown to regulate Prox1, which also is associated with EMT regulation [53]. In addition, Prox1 has been reported to have a strong association with diabetes [54–56]. miR-181a is a target for Fatty Acid Desaturase 1 (FADS1), a prominent genetic hit in AMP-Portal (http://www.type2diabetesgenetics.org). miR-205 is markedly downregulated in cells that have undergone EMT in response to transforming growth factor (TGF)- β or to ectopic expression of the protein tyrosine phosphatase Pez [57]. Inhibition of the miRNAs (miR-205 and others) was sufficient to induce EMT in a process requiring upregulation of ZEB1 and/or SIP1 [57]. ZEB1 represses E-cadherin [57] and induces expression of BMP antagonists such as Noggin and chordin-like 1 (CRDL1, CHRDL1) [58], which were highly ranked at baseline and follow-up, respectively. miR-497, the only prognostic miRNA with higher expression in cases, has higher expression levels in type 2 diabetes Goto-Kakizaki (GK) *vs* Wistar control rat islets [59]. In muscle cells, *Igfr* and *Insr* genes are direct targets of miR-497, which downregulates their expression [60]. Interestingly, miR-497 expression has also been shown to inhibit EMT in breast carcinoma by targeting Slug [61].

Until recently, most EMT regulation in the pancreas has been described in either the developing pancreas or regeneration models, such that β-cells become dedifferentiated and revert to a progenitor-like stage [62]. There is also evidence that rodent β-cells can partly convert to other endocrine cell types. By using a new marker for endocrine progenitor cells, aldehyde dehydrogenase 1A3 (ALDH1A3), Cinti *et al*. [63] recently showed a three-fold increase in cells positive for this marker and negative hormone staining in islets from type 2 diabetes donors compared to healthy controls. Islets from those with type 2 diabetes expressed β-cell-specific markers mislocalized to α-cells and δ-cells. Another recent study linked β-cell glucose sensitivity to insulin/glucagon bihormonal cells in nondiabetic insulin resistant humans [64].

Two other high-ranking proteins worthy of attention are endocan and carbonic anhydrase 3 (CA3). Endocan (also known as endothelial-cell specific molecule-1, ESM-1) identified here as lower in cases at both baseline and follow-up, is a soluble proteoglycan secreted by vascular endothelial cells. In adipocytes, endocan production is inhibited by insulin and cortisol, and circulating endocan levels have been found to be reduced in overweight and obese women [65], in insulin resistant [66] and in type 2 diabetes patients [67]. Endocan is also involved in other diseases such as chronic kidney disease, hypertension, and coronary artery disease [68]. Besides CA3, we observe several carbonic anhydrases (CA4 prognostic, CA3 and CA6 diagnostic) that show differential expression in cases *vs* controls. These enzymes regulate intracellular pH, and decreased CA3 levels have also been proposed to play a role in muscle fatigue and to lead to decreased ATP synthesis in mitochondria [69]. Obese animal models have decreased CA3 activity and concentration in white adipose tissue [21, 70].

With regard to pro-inflammatory biomarkers, higher baseline interleukin-6 (IL-6) levels have been reported to be associated with increased fasting insulin, insulin resistance, and β-cell function in the nondiabetic Whitehall II study cohort, while higher baseline adiponectin levels were associated with decrements in fasting glucose and insulin [71]. In our analysis, IL-6 was a univariate prognostic predictor in cases *vs* controls (Table 3). Other pro-inflammatory proteins emerging from our data were sTNFR2 and CD30, which are part of the TNF family (sTNFR2 up and CD30 down). TNFα signaling causes free fatty acid levels to rise via increased adipocyte lipolysis and downregulation of insulin signaling [72]. Increased sTNFR2 has been found in first-degree relatives of insulin resistant diabetic subjects [73] and in children with IGT [74], and was independently associated with increased aortic stiffness and pulse wave velocity in type 2 diabetes [75]. Both sTNFR1 and sTNFR2 have been shown to predict progression of kidney disease in both type 1 and type 2 diabetes [76, 77].

In summary, the current study is the first to have measured a large number of proteins and miRNAs in a case-control cohort of nondiabetic subjects matched not only on anthropometrics but also on the basis of careful physiological measurements of glucose tolerance, *i*.*e*., insulin sensitivity and β-cell function. Our results show that low adiponectin is strongly associated with β-cell dysfunction and is the top predictor of incident IGT. In addition, several proteins and miRNAs in the EMT pathway predicted a decline in β-cell function and glucose tolerance. While this biomarker discovery approach requires further validation in independent clinical cohorts, our data strongly suggest that the EMT pathway may in the near future provide useful biomarkers of β-cell function (and its decline) as well as potential new therapeutic targets. Novel circulating biomarkers that reflect or predict β-cell function could transform the way we treat type 2 diabetes, allowing for a more targeted and personalized approach, and potentially contribute to preventing its development entirely.

## Supporting information

S1 Table Diagnostic predictors of β-cell function. Fold change (FC) is calculated as Case—Control.
(DOCX)

S2 Table Prognostic predictors of β-cell function. Fold change (FC) is calculated as Case—Control.
(DOCX)

S3 Table Combined predictors of β-cell function. (DOCX)

S1 Objective 1 Protein data. (CSV)

S2 Objective 1 miRNA data. (CSV)

S3 Objective 1 Combined clinical, miRNA and protein data. (CSV)

S4 Objective 2 Protein data. (CSV)

S5 Objective 2 miRNA data. (CSV)

S6 Objective 2 Combined clinical, miRNA and protein data. (CSV)
