# Supplementary material for: Identification of novel biomarkers to monitor β-cell function and enable early detection of type 2 diabetes risk
Source: PLoS One. 2017 Aug 28;12(8):e0182932. doi: 10.1371/journal.pone.0182932 (PMC5573304; doi:10.1371/journal.pone.0182932)
Supplement: S1 Table — Fold change (FC) is calculated as Case—Control. (DOCX) [file pone.0182932.s001.docx]

## S1 Table. Diagnostic predictors of β-cell function.*

| Proteins | | | | miRNA | |
| --- | --- | --- | --- | --- | --- |
| **Univariate** | **FC** | **Multivariate** | **FC** | **Univariate** | **FC** |
| Fibronectin | 1.41 | FN1.3 | 1.24 | miR-21 | 0.32 |
| SCF.sR | 0.89 | SHBG | 0.76 | miR-145 | 0.37 |
| Carbonic.anhydrase.6 | 0.75 | Sialoadhesin | 1.22 | miR-151-3p | 0.37 |
| CD30 | 0.92 | G.CSF.R | 0.86 | miR-134 | 0.49 |
| Coagulation.Factor.XI | 0.91 | Growth.hormone.receptor | 1.14 | miR-215 | 0.32 |
| SHBG | 0.76 | KIRR3 | 1.15 | MIR-590-3P | 0.64 |
| FCGR1 | 0.85 | GPC2 | 0.86 | miR-485-3p | 0.44 |
| Nogo.Receptor | 1.13 | RUXF | 1.12 |  |  |
| Sialoadhesin | 1.22 | MMP.17 | 0.88 |  |  |
| sLeptin.R | 1.20 | VEGF121 | 1.10 |  |  |
| ULBP.2 | 1.20 | ILT.4 | 1.07 |  |  |
| MAPK5 | 0.64 | PECAM.1 | 0.95 |  |  |
| Olfactomedin.4 | 0.37 |  |  |  |  |
| PSA.ACT | 0.90 |  |  |  |  |
| IGFBP.4 | 1.11 |  |  |  |  |
| TNF.sR.II | 1.08 |  |  |  |  |
| IL.20 | 0.93 |  |  |  |  |
| Factor.I | 1.06 |  |  |  |  |
| FCG3B | 1.12 |  |  |  |  |
| CHL1 | 0.92 |  |  |  |  |
| MOZ | 0.91 |  |  |  |  |

* Fold change (FC) is calculated as Case – Control.
