# Supplementary material for: Identification of novel biomarkers to monitor β-cell function and enable early detection of type 2 diabetes risk
Source: PLoS One. 2017 Aug 28;12(8):e0182932. doi: 10.1371/journal.pone.0182932 (PMC5573304; doi:10.1371/journal.pone.0182932)
Supplement: S2 Table — Fold change (FC) is calculated as Case—Control. (DOCX) [file pone.0182932.s002.docx]

## S2 Table. Prognostic predictors of β-cell function.*

| **Proteins** | | | |
| --- | --- | --- | --- |
| **Univariate** | **FC** | **Multivariate** | **FC** |
| Transferrin | 0.93 | GDF2 | 0.77 |
| C1QBP | 0.93 | Carbonic.Anhydrase.IV | 0.91 |
| DC.SIGNR | 0.91 | IL.17D | 0.90 |
| Prekallikrein | 0.91 | CLC7A | 0.80 |
| PSMA | 0.92 | IL.6 | 1.27 |
| TMA | 0.92 | Desmoglein.1 | 0.93 |
| contactin.1 | 0.91 | Cadherin.E | 0.93 |
| Desmoglein.1 | 0.93 | Activin.RIB | 0.90 |
| TCCR | 0.89 | vWF | 0.77 |
| Cadherin.E | 0.93 | a1.Antitrypsin | 0.88 |
| RAD51 | 0.90 |  |  |
| IL.1b | 0.80 |  |  |
| CRDL1 | 0.91 |  |  |
| sFRP.3 | 0.92 |  |  |
| C34.gp41.HIV.Fragment | 0.88 |  |  |
| Mn.SOD | 0.89 |  |  |
| PDE9A | 0.88 |  |  |
| MM2 | 0.90 |  |  |
| PPIB | 0.88 |  |  |
| ATS13 | 0.85 |  |  |
| HRG | 0.88 |  |  |
| EphB6 | 0.94 |  |  |
| a1.Antitrypsin | 0.88 |  |  |
| Carbonic.anhydrase.9 | 0.83 |  |  |
| FCG2A.B | 1.30 |  |  |
| STK16 | 0.90 |  |  |
| CLC7A | 0.80 |  |  |
| Carbonic.Anhydrase.X | 0.84 |  |  |
| IGFBP.2 | 0.85 |  |  |
| PIK3CA.PIK3R1 | 1.11 |  |  |
| PlGF | 1.18 |  |  |
| Layilin | 0.91 |  |  |
| LCMT1 | 0.94 |  |  |
| sICAM.1 | 0.85 |  |  |
| CD23 | 0.86 |  |  |
| Sialoadhesin | 1.20 |  |  |
| a1.Antichymotrypsin | 0.94 |  |  |
| Cathepsin.V | 0.85 |  |  |
| Fibrinogen.g.chain.dimer | 0.90 |  |  |
| GRN | 0.93 |  |  |

* Fold change (FC) is calculated as Case – Control.
