# Supplementary material for: Identification of novel biomarkers to monitor β-cell function and enable early detection of type 2 diabetes risk
Source: PLoS One. 2017 Aug 28;12(8):e0182932. doi: 10.1371/journal.pone.0182932 (PMC5573304; doi:10.1371/journal.pone.0182932)
Supplement: S3 Table — (DOCX) [file pone.0182932.s003.docx]

## S3 Table. Combined predictors of β-cell function. *

| **Diagnostic** | | | **Predictive** | | |
| --- | --- | --- | --- | --- | --- |
| **Multivariate** | **FC** | **p-value** | **Multivariate** | **FC** | **p-value** |
| y3gluO120 | 1.56 | 1.12E-18 | Delta2hrIns | NA | 4.19E-05 |
| y3gluO90 | 1.46 | 8.96E-13 | Adiponectin | 0.74 | 0.001589616 |
| y3gluO60 | 1.30 | 8.78E-07 | miR-342-3p | 0.24 | 0.002327705 |
| y3insO120c | 1.98 | 0.000335056 | Deltapotentiation | NA | 0.002315145 |
| y3ogisrisc | 0.77 | 1.84E-05 | FGF-12 | 1.07 | 0.204526624 |
| Adiponectin | 0.75 | 0.000969956 | Cathepsin D | 0.91 | 0.002517203 |
| kallikrein.5 | 1.16 | 0.00030829 | STK16 | 0.89 | 0.033641869 |
| Carbonic.anhydrase.III | 1.41 | 0.046336585 | DLL4 | 0.81 | 0.012748741 |
| TNF.sR.II | 1.08 | 0.045313724 | TNFSF18 (CD30) | 0.96 | 0.207458868 |
| Cadherin.12 | 0.73 | 0.019638016 | miR-181a | 0.23 | 0.010660938 |
| Endocan | 0.85 | 0.003934917 | CDK5-p35 | 0.91 | 0.005352902 |
| y3nefa | 1.35 | 0.00064506 | NCAM-L1 | 0.88 | 0.007502453 |
| CRDL1 | 0.88 | 0.001521935 | MIR-590-3P | 0.58 | 0.016510402 |
| miR-181a | 0.17 | 0.003551795 | C1QBP | 0.92 | 0.017715444 |
| y3PFR | 0.66 | 0.005636231 | IL-11 | 0.86 | 0.051444911 |
| MIR-151-5P | 0.43 | 0.007235012 | IL-6 | 1.28 | 0.015318974 |
| G.CSF.R | 0.85 | 0.013531673 | Carbonic anhydrase 9 | 0.83 | 0.039524958 |
| miR-324-5p | 1.69 | 0.015011649 |  |  |  |
| FCG2A.B | 1.40 | 0.021093171 |  |  |  |
| miR-323-3p | 0.35 | 0.003439577 |  |  |  |

* Fold change (FC) is calculated as Case – Control.
